# Adipokinetic hormone (AKH), energy budget and their effect on feeding and gustatory processes of foraging honey bees

**DOI:** 10.1038/s41598-021-97851-x

**Published:** 2021-09-15

**Authors:** Gabriela de Brito Sanchez, Anna Expósito Muñoz, Li Chen, Weifone Huang, Songkun Su, Martin Giurfa

**Affiliations:** 1 College of Animal Sciences (College of Bee Science), Fujian Agriculture and Forestry University, Fuzhou, 350002 China; 2 Research Centre on Animal Cognition, Center for Integrative Biology, CNRS, University Paul Sabatier Toulouse III, 118 route de Narbonne, 31062 Toulouse Cedex 09, France; 3 Institut Universitaire de France (IUF), Paris, France

**Keywords:** Neuroscience, Physiology, Zoology

## Abstract

The adipokinetic hormone (AKH) of insects is considered an equivalent of the mammalian hormone glucagon as it induces fast mobilization of carbohydrates and lipids from the fat body upon starvation. Yet, in foraging honey bees, which lack fat body storage for carbohydrates, it was suggested that AKH may have lost its original function. Here we manipulated the energy budget of bee foragers to determine the effect of AKH on appetitive responses. As AKH participates in a cascade leading to acceptance of unpalatable substances in starved *Drosophila*, we also assessed its effect on foragers presented with sucrose solution spiked with salicin. Starved and partially-fed bees were topically exposed with different doses of AKH to determine if this hormone modifies food ingestion and sucrose responsiveness. We found a significant effect of the energy budget (i.e. starved vs. partially-fed) on the decision to ingest or respond to both pure sucrose solution and sucrose solution spiked with salicin, but no effect of AKH per se. These results are consistent with a loss of function of AKH in honey bee foragers, in accordance with a social life that implies storing energy resources in the hive, in amounts that exceed individual needs.

## Introduction

In mammals, the hormones glucagon and insulin play a key role for regulating blood glucose levels^1^. These peptide hormones are synthesized by endocrine glands in the pancreas and released into the blood, in response to changes in sugar levels. In target tissues such as the liver, they activate opposing metabolic pathways (e.g. glycogen breakdown by glucagon and glycogen synthesis by insulin), thereby maintaining steady-state glucose levels^2^.

Insects also possess peptide hormones that are essential for regulating both carbohydrates circulating in the hemolymph and stored lipids and glycogen in the fat body^3^, an organ that has been compared to the adipose tissue and liver due to its role in energy storage, utilization, and detoxification^4^. One of these hormones, the adipokinetic hormone (AKH)^5^, is secreted by the *corpora cardiaca* (CC) and elicits both carbohydrate and lipid mobilization from the fat body (trehalose from glycogen and diacylglycerol from triacylglycerol, respectively), acting as a functional homolog of glucagon^6,7^. This hormone signals to the G-protein coupled receptor encoded by the AKH receptor gene *AkhR* to elevate hemolymph lipid and/or trehalose titers, and thus redirects energy to the sites and processes where it is required^8,9^.

AKH was first identified in the locust *Locusta migratoria*^5,10^ where it stimulates lipolysis and locomotor activity essential for flight. Peptides with adipokinetic (and usually carbohydrate-mobilizing) potency have been demonstrated in various insects, including the desert locust *Schistocerca gregaria,* the tobacco hornworm *Manduca sexta,* the monarch butterfly *Danaus plexippus* and the cockroach *Periplaneta Americana*^11^, among others. In the adult fruit fly *Drosophila melanogaster*, AKH and the insulin-like peptides (DILPs) constitute an energy regulating system that is similar to the mammalian insulin-glucagon system^12,13^. For instance, starvation induces AKH release into the hemolymph to signal hunger^13^ and, consistently, experiments using transgenic manipulations of the *dAkh* gene showed that AKH induces both hypertrehalosemia and hyperlipemia^8,9^. In addition, CRISPR/Cas9 studies that allowed creating AKH *Drosophila* mutants showed that AKH regulates fat body content and hemolymph sugar levels as well as nutritional and oxidative stress responses^14^. Lipids seem to be the main energy reserve in *Drosophila* where lipid metabolism and levels are under the control of AKH, as shown by *Drosophila* mutants with AKH deficiency^15^. In contrast, glycogen seems to have a less significant role as a storage substance in flies, as shown by its lower levels and the fact that it is only under partial control of AKH. Thus, when the stores of glycogen are exhausted, carbohydrates might be synthesized from other sources^15^.

AKH has another interesting effect on food-ingestion decisions in the fruit fly. In flies, as in other species, starvation decreases sensitivity to unpalatable and potentially toxic compounds such as bitter tastants. Thus, starved flies are more prone to ingest bitter compounds, which would be otherwise rejected^16,17^. Such a decrease in bitter sensitivity is mediated by a pathway in which AKH neuroendocrine cells act either directly or indirectly on neurons expressing the short neuropeptide F (sNPF). These neurons act in turn on GABAergic neurons expressing the sNPF receptor, which inhibit bitter sensing neurons, decreasing thereby bitter sensitivity^16^.

The honey bee *Apis mellifera* constitutes an interesting case for the study of AKH, in particular in the case of honey bee foragers, as foraging activities are shaped by colony rather than by individual needs^18^. Foragers do not collect food for individual consumption but bring it back to the hive, where it is processed, stored and distributed^19–21^. Resources are stored within the colony in pollen and honey cells, in amounts that exceed largely individual requirements. Importantly, and in contrast to most solitary insects, honey bee foragers, which are the older bees in the colony, have a reduced fat body^22^ with low glycogen reserves compared to younger nest bees^23^ and experience a significant reduction in abdominal lipids in the transition that precedes the onset of foraging^24^. To sustain the energetic demands of foraging flights, forager bees load in the crop minute amounts of honey before departing from the hive, which serve as fuel for the rest of the foraging bout^21,25,26^. As long as sugar is present in their crops, bees keep flying for food; yet, they stop flying as crop carbohydrates disappear^27^. They thus rely on sugar available in the collective stores of the hive rather than on fat body resources to sustain their foraging activities^23^. These physiological and behavioral specificities indicate that the social life of bees has important consequences for the regulation of their metabolic pathways.

The first report on AKH in bees was provided by Lorenz et al.^28^, who detected its presence in the CC of *Apis mellifera ligustica (Aml)* but not of *Apis mellifera carnica* (*Amc*). Extracts of *Aml* CC elicited an adipokinetic/hypertrehalosemic response when injected into crickets and cockroaches, but extracts of *Amc* CC did not elicit such responses. As the *Aml* AKH has the same structure as the AKH of *Manduca sexta*^28^, the latter was used to inject winter and spring/summer bees of both races. A weak hypertrehalosemic response to AKH injection was observed in winter *Aml* and *Amc* bees, which have more developed fat bodies than active spring/summer bees. In the latter, no response to AKH injection was detected. It was concluded that although AKH is present in the CC of bees, it does not have a glycogen-mobilizing function in active foragers, which lack of an adequate glycogen store in their fat body for its effective utilization^28^. Along the same line of reasoning, it was suggested that AKH might have lost its original function in social bees^29^. Importantly, although the physiological measurements performed in these various works support this hypothesis, analyses of feeding responses of honey bee foragers in response to variations of AKH levels and energy budget are still missing.

Honey bee AKH arouses further interest in the light of its effect on the acceptance of unpalatable food upon starvation. In fruit flies, which ingest bitter compounds that they would otherwise reject when they are hungry^16,17^, this acceptance is thought to depend on AKH, which activates a cascade leading to bitter-receptor inhibition, and thus to the acceptance of bitter food^16^. In honey bees, the existence of bitter receptors is controversial^30–32^, and the rejection of sucrose solution spiked with bitter substances was explained by the inhibition that these substances produce on sucrose receptors^33^, which leads to a devaluation of bitter-sweet solutions^30,33^. As bees, may indeed ingest unpalatable substances under specific circumstances^34,35^, it is worth asking if AKH is also involved in this food acceptance, in particular, under the eventual absence of bitter receptors. If this were the case, bees with higher AKH levels would be more prone to accept mixtures of sweet and bitter tastants as a consequence of a molecular cascade that would impair sugar-receptor inhibition by bitter substances rather than via a direct inhibition of bitter receptors. Alternatively, if AKH has lost its original function in forager bees, no effect should be seen at the level of unpalatable food acceptance.

Here we manipulated both the energy budget of honey bee foragers and AKH levels via topical application of different doses of AKH on the bee thorax^36^. AKH was topically administered to starved and partially fed forager bees to test different predictions on the possible effects of this hormone. In starved foragers, with an empty crop, low glycogen reserves and reduced fat body^22,23^, AKH would not have a significant effect on appetitive behavior other than that induced by hunger itself. As there would be less internal resources to mobilize and their crop would be empty, enhancing AKH levels would not modify significantly feeding behavior. On the contrary, in partially fed bees, which have more energetic resources to mobilize given the presence of food in their crop, AKH might induce a decrease in feeding responses beyond the limits set by the feeding state itself, if the hormone conserves a functional role for the regulation of the bees’ energy budget. In addition, by studying the responses of starved and partially fed bees treated with AKH to sucrose solution spiked with salicin, we also tested the effect of this hormone on unpalatable-food acceptance. Previous works have showed that this mixture has a reduced appetitive attraction for honey bees^33–35^. Thus, if AKH is still functional and plays a similar role as in fruit flies, it should increase the consumption of the sucrose-salicin solution in AKH-treated foragers via a cascade acting on gustatory receptors.

To test these hypotheses, we quantified two types of appetitive response: the proboscis extension response (PER), which is triggered by contact of sweet tastants with antennal sucrose receptors^37,38^, and individual food consumption. We thus determined the effect of both AKH and energy budget on gustatory and feeding processes of bees presented with either pure sucrose solution or sucrose solution spiked with the bitter substance salicin.

## Materials and methods

**Figure 1:**
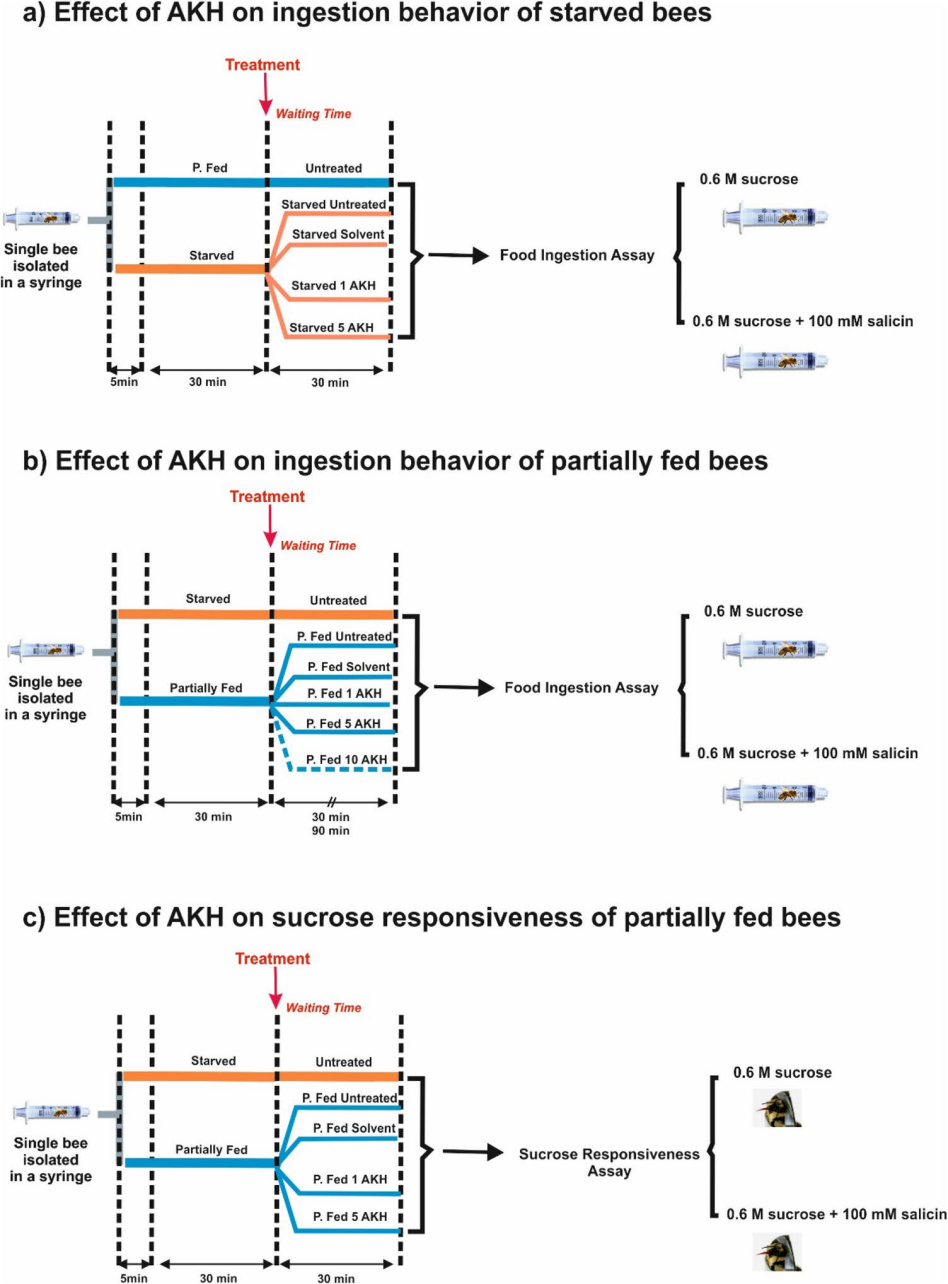
Experimental protocols applied to partially fed and starved bees. Honey bee foragers were enclosed in individual syringes during 5 min. They then received two feeding treatments during the next 30 min: starved bees (‘Starved’) received no food while partially fed bees (‘P. Fed’) received 5 µl of honeydew (a mixture of 10 ml of distilled water, 30 g of honey, 5 g of pollen and 5 g of sugar) and then 20 µl of 1.8 M sucrose solution. (**a**) Effect of AKH on food-ingestion of starved bees. After topical application and an additional resting period of 30 min, bees were maintained in their syringes and food was delivered by means of a 100–200 µl Eppendorf tip inserted in the syringe during a 1-h period. Four groups of starved bees and one group of partially fed bees were run in parallel. The group of partially fed bees (‘P. Fed’) and one group of starved bees (‘Starved’) remained untreated (controls). The remaining three groups of starved bees were subjected to different treatments: one was topically exposed with the solvent used as vehicle for AKH (‘Starved Solvent’) while the other two were topically exposed with either 1 µg/µl (‘Starved 1 AKH’) or 5 µg/µl (‘Starved 5 AKH’) of AKH solution. Bees were offered either 100 µl of a 20% (0.6 M) pure sucrose solution or 100 µl of a mixture of 20% (0.6 M) sucrose solution and 100 mM salicin. (**b**) Effect of AKH on food-ingestion of partially fed bees. After topical application and an additional resting period of either 30 min or 90 min, bees were maintained in their syringes and food was delivered by means of a 100–200 µl Eppendorf tip inserted in the syringe during a 1-h period. Six groups of bees were established for the 30-min resting period (Starved, P. Fed, P. Fed Solvent, P. Fed 1 AKH, P. Fed 5 AKH and P. Fed 10 AKH). Five groups were used for the 90-min resting period (Starved, P. Fed, P. Fed Solvent, P. Fed 1 AKH and P. Fed 5 AKH). Bees were fed with either 100 µl of a 20% (0.6 M) pure sucrose solution or 100 µl of a mixture of 20% (0.6 M) sucrose solution and 100 mM salicin. (**c**) Effect of AKH on sucrose responsiveness of partially fed bees. After topical application and an additional resting period of 30 min, bees were subjected to a sucrose-responsiveness assay in which their proboscis extension response (PER) was quantified upon antennal stimulation with a series of increasing concentrations of pure sucrose solution (0.1%, 0.3%, 1%, 3%, 10% and 30% w/w) or the same solutions spiked with 100 mM salicin. Five groups were used: Starved, P. Fed, P. Fed Solvent, P. Fed 1 AKH and P. Fed 5 AKH.

Experiments were performed during springtime days (April–May), when forager bees were active. Honey bee foragers *Apis mellifera ligustica* were caught in the morning upon landing at a feeder containing a 1 M sucrose solution to which they were previously trained. Capture occurred before the bees started feeding to ensure that their crop was empty at the start of the experiments and that their appetitive state was comparable. Upon capture, bees were enclosed individually within 5 ml syringes and brought to the laboratory (Fig. 1).

### Feeding treatments

Bees were enclosed in their respective syringes during 5 min and then divided in two groups that received two feeding treatments during the next 30 min (Fig. 1). Starved bees received no food during that period. Partially fed bees received 5 µl of honeydew (a mixture of 10 ml of distilled water, 30 g of honey, 5 g of pollen and 5 g of sugar) and then 15 µl of 50% (1.8 M) sucrose solution via 100–200 µl Eppendorf tips, which could be inserted in the syringe hub^34^. In total, 20 µl of food were provided to these bees, which represents one third of their average crop full capacity^39^. The 5 µl of honeydew represent a strong energetic supplement. Yet, its high viscosity prevents rapid consumption of larger volumes, even by adding water to it. We thus provided a small amount (5 µl) of this food, which bees easily consumed, and complemented it with the 15 µl of concentrated sucrose solution (50%), which bees ingested without problems. Bees could move within the syringe and decide to eat or not the food provided in the Eppendorf tip. These enclosing conditions were chosen as they enhance the bees’ predisposition to ingest less attractive food^34^.

### Quantification of energy budgets

To verify that starved and partially fed bees differed in their energy budget at the end of the 30-min period, we collected 2 µl of their hemolymph by puncturing the neck membrane, and quantified their hemolymph sugar levels using the anthrone colorimetric kit for trehalose (Solarbio, Beijing, #BC0330), following manufacture instructions. These bees were not used further for behavioral experiments. They allowed us to verify that our treatment resulted in different energy budgets in starved and partially fed bees. We focused on trehalose as it is the principal sugar circulating in the hemolymph of most insects^40^. In the honey bee, the hemolymph concentration of trehalose is important in the feedback mechanism that regulates the activity of sugar transport through the proventriculus, and thus of the bee metabolic rate^41^.

### AKH treatment

To manipulate AKH levels, we performed a topical application of this hormone on the thorax of starved and partially fed foraging bees, i.e. bees that had spent 30 min in their individual syringes, without or with food access, respectively (see above). Both types of foragers differed only in their feeding state. We predicted that no modulation of appetitive responses should be observed in starved foragers following AKH topical application, as these bees do not dispose of significant resources to mobilize. On the contrary, if AKH is still functional, it should decrease the appetitive responses of partially fed bees, which possess more energetic resources to mobilize in the crop than starved bees.

*Apis mellifera* AKH^42^ was purchased from GenScript (NJ, USA). It was dissolved in a mixture of 20% DMSO and 80% acetone to obtain the concentrations of 1, 5 and 10 µg/µl. The resulting solutions were delivered to the dorsal thorax of bees by means of a pipette, taking care that it did not spread into the neck, the petiole or around the wing hinges^36^. The topical application procedure ensures efficient penetration through the bee cuticula^36^ and has been employed successfully for diverse molecules, including pesticides, herbicides, fungicides^43–45^ and other peptides (de Brito Sanchez et al., unpublished). The different doses of AKH allowed us to study potential dose–response effects for both food ingestion and sucrose responsiveness (see below).

After topical application, bees were held in their syringes for further 30 min or 90 min, depending on the experiment, to allow the solvent to penetrate the cuticle (Fig. 1). For starved foragers, this represents one or one and half-hour without food access, respectively, a considerable period during which they would have performed 14.57 ± 0.31 (n = 30) or 22.24 ± 0.20 (n = 30) foraging bouts to the feeder at which they were captured. The effect of AKH was evaluated at these times because if it acts as the equivalent of the mammalian glucagon in the case of foragers, it should promote relatively fast mobilization of energetic resources^46^.

### Behavioral assays

We determined if AKH modulates sucrose responsiveness and food ingestion in starved and partially fed foraging bees confronted with a 0.6 M (20% w/w) pure sucrose solution or with the same sucrose solution spiked with 100 mM salicin. Using the mixture of sucrose solution and salicin, allowed us to test if AKH increases the acceptance of unpalatable food given the reduced attractiveness of this mixture^33–35^. Sucrose solutions were prepared using sucrose of analytical grade (Sigma-Aldrich, France) diluted in purified and deionized water (Milli-Q system, Millipore, Bedford, USA). Salicin was also obtained from Sigma-Aldrich (France).

### Food ingestion assay

After topical application and the subsequent resting period, bees were maintained in their syringes and food was delivered by means of a 100–200 µl Eppendorf tip inserted in the syringe hub during a 1-h period. Each bee received either 100 µl of the pure sucrose solution or 100 µl of the mixture of sucrose solution and salicin. The amount of solution consumed by each bee was measured as the difference between the original volume of 100 µl and the remaining volume measured by the pipette. Consumption values were corrected for evaporation, which was measured in empty control syringes^47^, and expressed in terms of µl consumed per bee. We tested the null hypothesis of a lack of difference in consumption between the different groups of bees presented with the pure sucrose solution or with the sucrose solution spiked with salicin.

### Sucrose responsiveness assay

After topical application and the subsequent resting period, bees were harnessed in individual standing tubes from which only the head protruded. They were then subjected to a standard assay for the quantification of sucrose responsiveness^48–51^. In this assay, proboscis extension responses (PER) to increasing concentrations of sucrose solution were quantified. Each bee was presented with six sucrose solutions of increasing concentrations (0.1%, 0.3%, 1%, 3%, 10%, and 30%; w/w). These solutions were delivered to both antennae with the help of a toothpick. In the case of the sucrose solution spiked with salicin, the same sucrose concentrations were used to which salicin was added. The inter-stimulus interval was 1 min.

Bees that did not respond to any sucrose concentration of the series were presented with a 50% (1.8 M) sucrose solution at the end of the sequence; those not responding even to this solution were excluded from successive analyses^49^. We also discarded bees responding to water, which we used to control for the effect of thirst on sucrose responsiveness^52^. Moreover, bees exhibiting inconsistent responses to sucrose (i.e. responding to a lower but not to a higher sucrose concentration) were also discarded, as preconized by the standard method of sucrose-responsiveness evaluation, because the lack of response to the higher concentration may be due to an uncontrolled motor problem and not to sucrose sensitivity itself^53^. The percentage of bees discarded in each sucrose responsiveness experiment did not exceed 5% of the total bees assayed.

For each bee retained for the analysis, an individual sucrose response score (SRS) was calculated as the number of sucrose concentrations eliciting a PER. SRS ranged from 0 to 6. Bees with a SRS of 0 did not respond to any concentration (but responded to the additional sucrose concentration of 50% delivered at the end of the sequence) while bees with a SRS of 6 responded to all six sucrose concentrations. We tested the null hypothesis of a lack of difference in sucrose responsiveness between the different groups of bees stimulated with increasing concentrations of either pure sucrose solution or sucrose solution spiked with salicin.

### Experimental groups

#### Effect of AKH on ingestion behavior of starved bees

Four groups of starved bees and one group of partially fed bees (see above for feeding treatments) were used to determine the effect of AKH on individual ingestion of starved bees (Fig. 1a). The group of partially fed bees (‘P. Fed’) and one group of starved bees (‘Starved’) remained untreated (controls). The remaining three groups of starved bees were subjected to different treatments: one was topically exposed with the solvent used as vehicle for AKH (‘Starved Solvent’) while the other two were topically exposed with either 1 µg/µl (‘Starved 1 AKH’) or 5 µg/µl (‘Starved 5 AKH’) of AKH solution.

Individuals of all groups spent 30 min within their corresponding syringes, and then 30 additional min following topical application or equivalent handling (untreated bees). This time was chosen as AKH is supposed to promote fast mobilization of energetic resources and thus provide energy in periods shorter than 60 min. Moreover, as the majority of bees in this experiment were starved, there was a high risk of inducing significant mortality through confinement in individual syringes for periods longer than 1 h.

After this confinement time, bees were subjected to an ingestion assay (see above) to measure their consumption of either a pure sucrose solution or a sucrose solution spiked with salicin.

#### Effect of AKH on ingestion behavior of partially fed bees

Partially fed bees and starved bees (see above for feeding treatments), and two resting periods of 30 min and 90 min post topical application were used (Fig. 1b). For the 30-min resting period, six groups of bees were established: one group of starved untreated bees (‘Starved’), one group of partially fed untreated bees (‘P. Fed’), one group of partially fed bees that was topically exposed with the solvent used as vehicle for AKH (‘P. Fed Solvent’), and three groups of partially fed bees that were topically exposed with either 1 µg/µl (‘P. Fed 1 AKH’), 5 µg/µl (‘P. Fed 5 AKH’) or 10 µg/µl (‘P. Fed 10 AKH’). For the 90-min resting period, five groups of bees were studied in parallel: Starved, P. Fed, P. Fed Solvent, P. Fed 1 AKH and P. Fed 5 AKH.

After their respective resting period, bees were subjected to an ingestion assay (see above) with either pure sucrose solution or sucrose solution spiked with salicin.

#### Effect of AKH on sucrose responsiveness of partially fed bees

Partially fed bees and starved bees were studied in parallel (see above for feeding treatments). A resting period of 30 min post topical application was used (Fig. 1c). Five groups of bees were established: one group of starved untreated bees (‘Starved’), one group of partially fed untreated bees (‘P. Fed’), one group of partially fed bees that was topically exposed with the solvent used as vehicle for AKH (‘P. Fed Solvent’), and two groups of partially fed bees that were topically exposed with either 1 µg/µl (‘P. Fed 1 AKH’) or 5 µg/µl (‘P. Fed 5 AKH’).

### Statistics

Sugar levels in hemolymph of starved and fed bees were compared using a Mann–Whitney test for independent samples, as data were not normally distributed. Food ingestion values (µl/bee) were analyzed by means of One-Factor Analysis of Variance (ANOVA) for independent groups, followed by post-hoc Tukey tests for between-group comparisons. If the assumptions of ANOVA were not met, a Kruskal Wallis test for comparing independent groups was used followed by a test for multiple comparison of mean ranks. Sucrose responses (PER: 1 or 0) of individual bees in the sucrose responsiveness assays were examined using a generalized linear mixed model (GLMM) with a binomial error structure—logit-link function-, glmer function of R package lme4^54^. ‘Response’ was entered as dependent variable, ‘treatment’ as fixed factor and ‘sucrose concentration’ as covariate. ‘Individual’ identity (ID) was considered as a random factor in order to allow for repeated measurements. We retained the significant model with the highest explanatory power (i.e. the lowest AIC value). We used Dunnett’s post-hoc tests (glht function from R package multcomp) to detect differences between the different groups^55^. Differences between the sucrose response scores (SRS) of different groups of bees were analyzed using a Kruskal–Wallis test followed by post hoc pairwise comparisons based on a Mann–Whitney U test with alpha value corrected according to the Holm–Bonferroni method. For all analyses, the alpha level was set to 0.05. All statistical analyses were performed with R 3.2.3^56^ or with Statistica 13.2 (StatSoft, Inc.).

## Results

### Energy budget of starved and partially fed bees before AKH treatment

**Figure 2:**
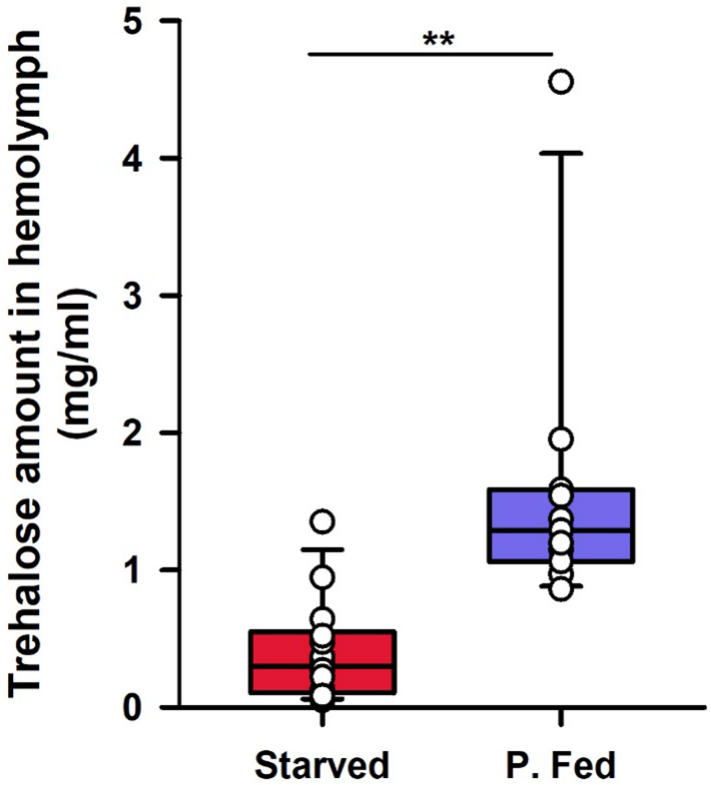
Energy budget of starved (‘Starved’) and partially fed (‘P. Fed’) bees before AKH treatment. Trehalose contents (mg) in hemolymph samples (ml) obtained from starved and fed bees before AKH treatment. The figure shows the median (horizontal line) and the 10th, 25th, 75th and 90th percentiles as vertical boxes with error bars and the corresponding overlaid scatter plots. Sugar levels were quantified using a colorimetric kit for trehalose (see text for details). **P < 0.001.

At the end of the 30-min period used to establish the two main categories of starved and partially fed bees, trehalose levels in hemolymph differed significantly (Fig. 2; Mann–Whitney test for independent samples; W_11,14_ = 7; P < 0.001). Fed bees had 4 times more sugar (1.59 ± 0.31 mg/ml, mean ± S.E., n = 11) than starved bees (0.40 ± 0.1 mg/ml, mean ± S.E., n = 14), thus showing that the feeding treatment was effective to induce differences in the energy budget of the two groups of bees.

### Effect of AKH on the ingestion behavior of starved bees

We first analyzed if AKH affected the decision of starved bees to ingest food. To this end, we compared the ingestion (µl/bee) of pure 0.6 M sucrose solution in five groups of bees: one group of partially fed bees (‘P. Fed’) and four groups of starved bees (‘Starved’). The partially fed bees and one group of starved bees remained untreated. The remaining three groups of starved bees were topically exposed with the solvent used as vehicle for AKH (‘Starved Solvent’) or with one of two doses of AKH (1 µg/µl: ‘Starved 1 AKH’, or 5 µg/µl: ‘Starved 5 AKH’).

**Figure 3:**
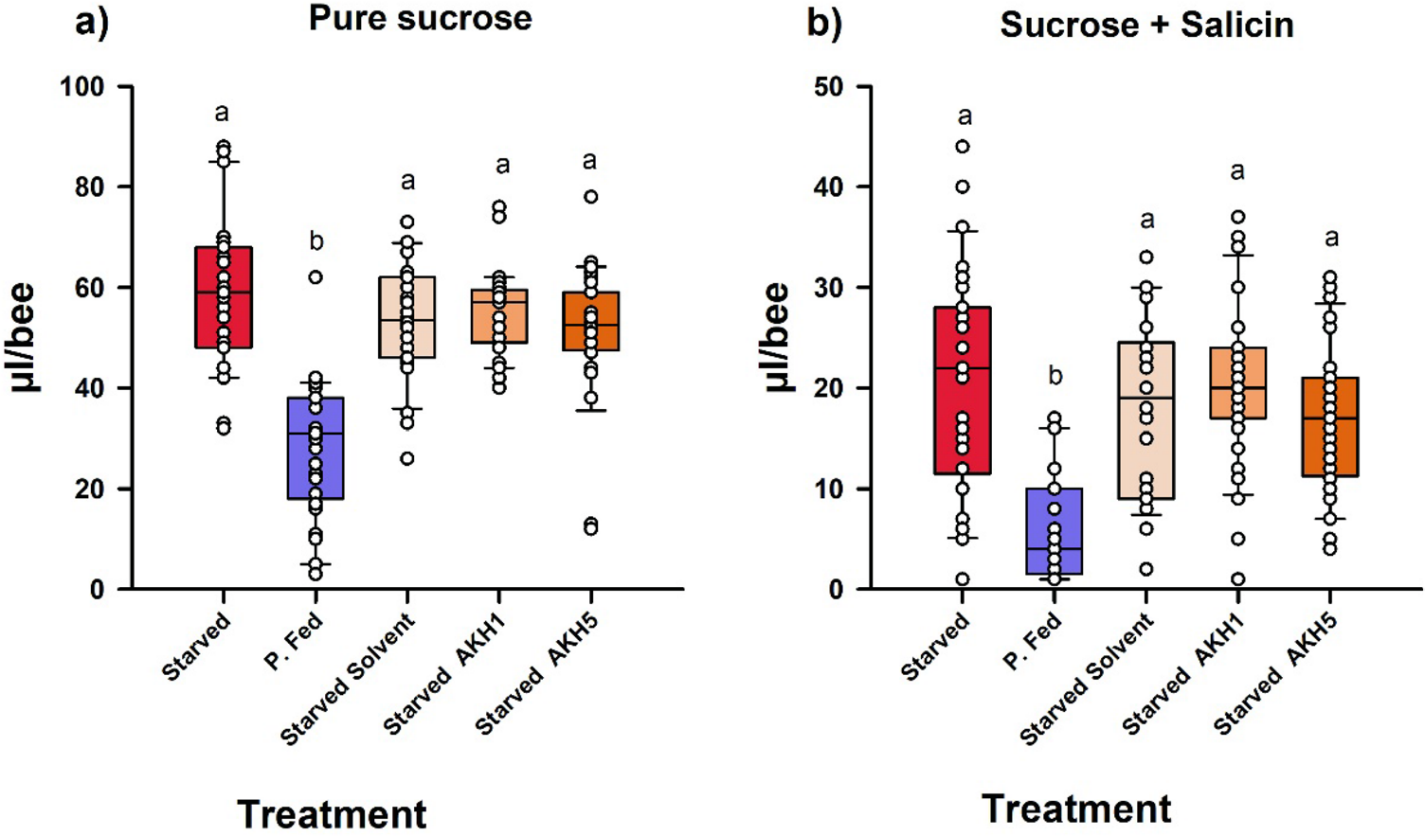
Ingestion (µl/bee) of starved foraging bees treated or not with AKH, when presented with a pure 0.6 M sucrose solution (**a**) or a 0.6 M sucrose solution spiked with 100 mM salicin (**b**). Food was provided 30 min after the topical treatment. Bees were either starved (‘Starved’) or partially fed (‘P. Fed’) with a honeydew diet. P. Fed bees and one group of Starved bees were left untreated. One group of starved bees was topically exposed with solvent (‘Starved solvent’). The other two groups of starved bees were topically exposed with AKH 1 µg/µl (‘Starved AKH1’) or AKH 5 µg/µl (‘Starved AKH5’). (**a**) Consumption of pure 0.6 M sucrose solution. Thirty min after topical treatment, starved bees consumed significantly more 0.6 M sucrose solution than partially fed bees (n = 29). Starved (n = 29), Starved solvent (n = 30), Starved AKH1 (n = 29) and Starved AKH5 bees (n = 28) did not differ in their consumption of sucrose solution. (**b**) Consumption of 0.6 M sucrose solution spiked with 100 mM salicin solution. Thirty min after topical treatment, starved bees consumed significantly more sucrose solution spiked with salicin than partially fed bees. Yet, consumption decreased significantly in all groups compared to the situation in which pure sucrose solution was offered (note the different ordinate scale in (**a,b**), which was changed to appreciate data dispersion), thus showing the unpalatable nature of the mixture offered. Partially fed bees (n = 29) consumed significantly less mixture than starved bees. Starved (n = 30), Starved solvent (n = 26), Starved AKH1 (n = 31) and Starved AKH5 bees (n = 32) did not differ in their consumption of sucrose solution. These results show that AKH did not affect the ingestion behavior of starved bees. In both panels, data shown are the median (horizontal line) and the 10th, 25th, 75th and 90th percentiles as vertical boxes with error bars and the corresponding overlaid scatter plots. Different letters above boxes indicate significant differences (P < 0.0001).

Thirty min after topical application, ingestion of pure sucrose solution varied significantly between groups (Fig. 3a; One-Factor ANOVA; F_4,139_ = 25.55; P < 0.0001). All groups of starved bees ingested significantly higher amounts of sucrose solution than the group of partially fed bees. The crop load capacity of the four starved groups was close to the average full crop capacity of 60 µl^39^ (54.52 ± 1.12 µl/bee; mean ± S.E., n = 116), thus showing that hungry bees tended to fill their crop irrespective of the AKH/solvent treatment received. The volume ingested by the starved groups did not differ significantly (Tukey test, P > 0.05 in all cases) while the volume ingested by the group of partially fed bees was significantly smaller than that of the starved groups (Tukey test, P < 0.0001 for all comparisons). This result shows that AKH did not affect the ingestion behavior of starved bees, either because it is not functional or because these bees lacked from enough energetic resources to mobilize in the absence of a developed fat body. The only factor introducing a difference in ingestion behavior was the feeding state: as expected, partially fed bees ingested less sucrose solution than starved bees.

When bees were offered a sucrose solution spiked with salicin (Fig. 3b), the volume ingested by all groups was less than half the value observed in the absence of salicin (compare with Fig. 3a), thus showing that this mixture has indeed a low palatability for honey bees. The volume ingested differed significantly between groups (One-Factor ANOVA; F_4,143_ = 15.46; P < 0.0001). As in the previous experiment, all groups of starved bees ingested significantly higher amounts of sucrose solution than the group of partially fed bees (Tukey test, P < 0.0001 for all comparisons). The starved groups did not differ between them (Tukey test, P > 0.05 for all comparisons) and their average consumption was 18.82 ± 0.82 µl/bee (mean ± S.E., n = 119) at the end of the experiment. These results confirm that AKH did not affect the ingestion behavior of starved bees and that the only factor introducing a difference in ingestion behavior between groups was the feeding state of bees. The mixture offered to the bees was clearly unpalatable as shown by the general reduction in consumption. Yet, contrary to what has been observed in fruit flies, AKH treatment did not enhance the consumption of this unpalatable mixture in the starved bees.

### Effect of AKH on the ingestion behavior of partially fed bees

We next determined if AKH affected the ingestion behavior of partially fed foraging bees, which contrary to starved bees, had energetic resources that could be mobilized by AKH if this hormone were functional. We first analyzed if consumption of a 0.6 M pure sucrose solution varied 30 or 90 min after AKH topical application.

**Figure 4:**
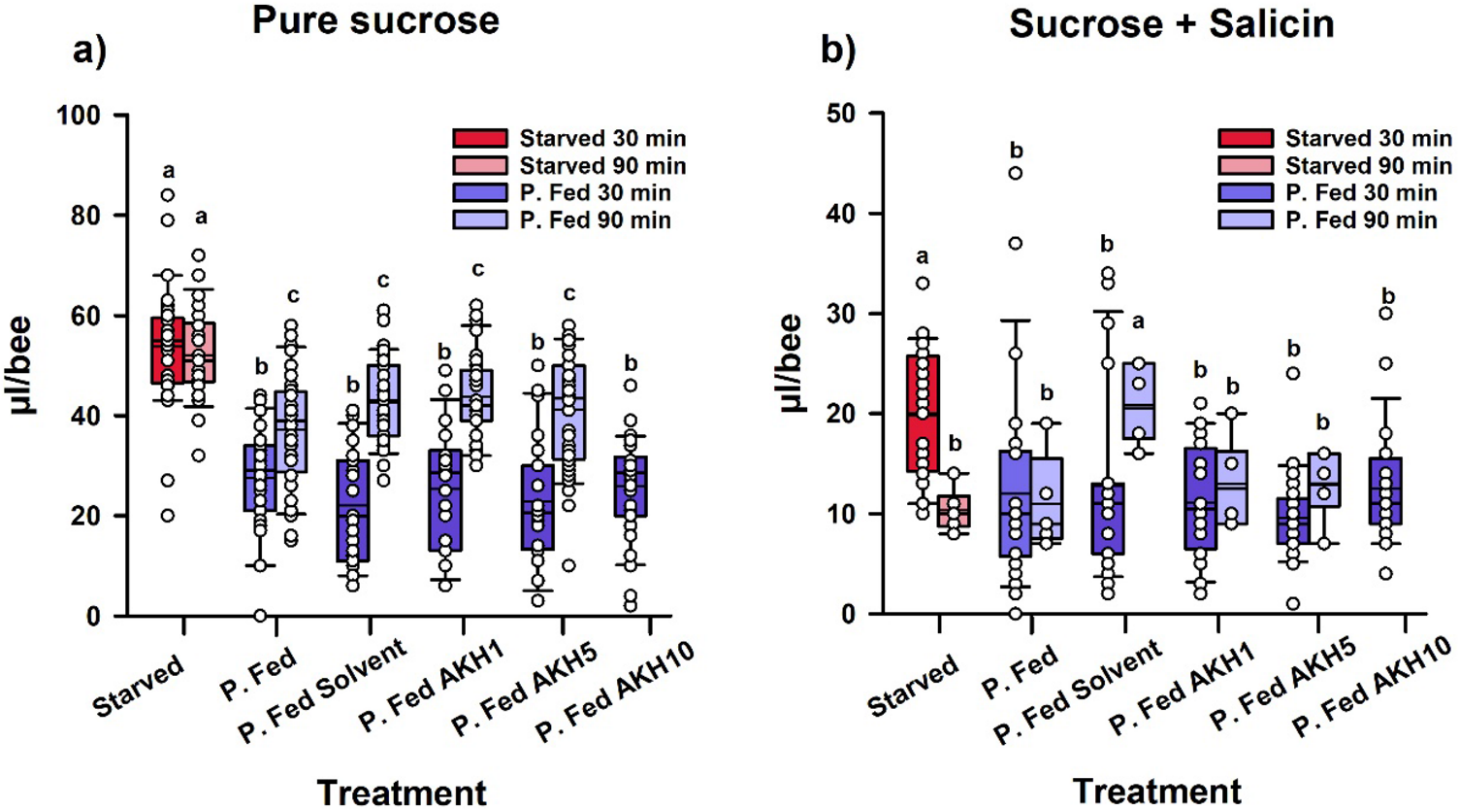
Ingestion (µl/bee) of partially fed foraging bees (‘P. Fed’) treated or not with AKH, when presented with a pure 0.6 M sucrose solution (**a**) or a 0.6 M sucrose solution spiked with 100 mM salicin (**b**). Food was provided 30 min or 90 min after the topical treatment. Bees were either starved (‘Starved’) or partially fed with a honeydew diet. Partially fed bees were left untreated (‘P. Fed’), topically exposed with solvent (‘P. Fed solvent’) or with AKH (either 1, 5 or 10 µg; ‘P. Fed AKH1’, ‘P. Fed AKH5’, or ‘P. FedAKH10’ respectively). (**a**) Consumption of pure 0.6 M sucrose solution. Thirty min after topical treatment, starved bees (red box; n = 29) consumed significantly more 0.6 M sucrose solution than partially fed bees (blue boxes). P. Fed (n = 27), P. Fed solvent (n = 27), P. Fed AKH1 (n = 22), P. Fed AKH5 (n = 24) and P. Fed AKH10 bees (n = 30) did not differ in their consumption of sucrose solution. Ninety min after topical treatment, food consumption increased in P. Fed bees (light-blue boxes), which had used probably part of their reserves, but the general response pattern remained the same: starved bees (pink box; n = 26) consumed significantly more 0.6 M sucrose solution than P. Fed bees. No significant differences were found between P. Fed (n = 32), P. Fed solvent (n = 37), P. Fed AKH1 (n = 34) and P. Fed AKH5 bees (n = 36). (**b**) Consumption of pure 0.6 M sucrose solution containing 100 mM salicin. Thirty min after topical treatment, starved bees (red box; n = 24) consumed significantly more 0.6 M sucrose solution spiked with salicin than P. Fed bees (blue boxes). P. Fed (n = 26), P. Fed solvent (n = 26), P. Fed AKH1 (n = 20), P. Fed AKH5 (n = 21) and P. Fed AKH10 bees (n = 24) did not differ in their consumption of sucrose solution spiked with salicin. Salicin reduced significantly the consumption of sucrose solution compared to pure sucrose solution (note the different ordinate scale in (**a,b**), which was changed to appreciate data dispersion) but no effect of AKH was detected. Ninety min after topical treatment, food consumption decreased in starved bees (pink box; n = 6), which did not differ from all P. Fed groups except for the P. Fed solvent group (n = 5 for P. Fed; n = 6 for P. Fed solvent, P. Fed AKH1 and P. Fed AKH5). In both panels, data shown are the median (horizontal line) and the 10th, 25th, 75th and 90th percentiles as vertical boxes with error bars and the corresponding overlaid scatter plots. Different letters above boxes indicate significant differences (P < 0.05).

Thirty min after topical application, ingestion of the pure sucrose solution varied significantly between groups (Fig. 4a; One-Factor ANOVA; F_5,153_ = 30.31; P < 0.0001). The group of starved bees ingested significantly larger amounts of sucrose solution (red box; 53.76 ± 2.36 µl/bee; mean ± S.E.; n = 29) than the partially fed groups (blue boxes; Tukey test, P < 0.001 in all cases). The crop load of starved bees was close to the average full-crop capacity of 60 µl^39^. All the five partially fed groups ingested smaller amounts of sucrose solution (average consumption 24.76 ± 1.00 µl/bee; mean ± S.E., n = 130), which did not differ significantly (Tukey tests, P > 0.05 in all cases), thus showing that neither the solvent nor the different doses of AKH affected food ingestion.

Ninety min after the topical treatment (Fig. 4a), ingestion also differed significantly between groups (F_4,160_ = 8.58; P < 0.0001). Starved bees exhibited a food consumption that was again close to the full-crop capacity (pink box; 52.11 ± 1.78 µl/bee; mean ± S.E., n = 26) and that was higher than that of the four partially fed groups (light-blue boxes; Tukey test, P < 0.001 in all cases). These groups doubled their food consumption with respect to the 30-min resting period (41.41 ± 0.87 µl/bee; mean ± S.E., n = 138) but did not differ significantly between them (Tukey tests, P > 0.05 in all cases).

A comparison between the 30-min and the 90-min resting period was possible for five groups (Starved, P. Fed, P. Fed Solvent, P. Fed AKH1 and P. Fed AKH5). It revealed a significant effect of the resting period (F_1,284_ = 103.17; P < 0.0001), the group (F_4,284_ = 38.63; P < 0.0001) and a significant interaction between resting period and group (F_4,284_ = 10.15; P < 0.0001). The starved groups did not differ between the two resting periods (Tukey test, P = 0.99), but the partially fed groups did (P < 0.05 for each within-group 30- vs. 90-min comparison). Thus, after a prolonged resting/starvation period, partially fed bees increased their food consumption but no effect of AKH was detected, neither on a dose nor on a time basis.

We then studied if AKH treatment enhanced the propensity of partially fed bees to consume the unpalatable sucrose solution spiked with salicin (Fig. 4b). Thirty min after topical treatment, bees also varied their consumption of the mixture depending on the treatment received (Kruskal Wallis test, H_5,141_ = 28.86; P < 0.0001). A multiple-comparison test between mean ranks showed that the starved group (Fig. 4b; red box) consumed significantly more sucrose solution with salicin than all fed groups (P < 0.05 for all comparisons). Consumption of the starved group (19.75 ± 4.03 µl/bee; mean ± S.E.; n = 24) was, however, reduced compared to the full crop of starved bees ingesting pure sucrose solution (see above and Fig. 4a), which confirms the low attraction of the mixture provided. Volumes ingested by all five partially fed groups were smaller (blue boxes; 11.70 ± 0.70 µl/bee; mean ± S.E.; n = 117) and did not differ significantly (Tukey tests, P > 0.05 in all cases). These results show again that only the feeding state determined the amount of solution ingested. The addition of salicin to the sucrose solution decreased ingestion, yet neither the solvent nor the different doses of AKH had an effect on it.

Ninety min after the topical treatment, food ingestion varied again between groups (Fig. 4b; F_4,22_ = 5.34; P < 0.01). This time, significance was introduced by the partially fed group treated with solvent only, which had a higher significantly consumption (light-blue box; 20.83 ± 8.51 µl/bee; mean ± S.E., n = 6) than all other groups (11.83 ± 0.77 µl/bee; mean ± S.E.; n = 23) (Tukey tests, P < 0.05 in all cases). Importantly, the two AKH-treated groups did not vary their food ingestion, which did not differ from that of the untreated P. fed group (P > 0.05 in both cases). No significant differences were found between the two resting times of 30 and 90 min (F_1,138_ = 0.03; P = 0.86) in accordance with the general reduction of food consumption induced by the less attractive mixture of sucrose and salicin solution.

### Effect of AKH on sucrose responsiveness of partially fed bees

We finally studied the capacity of AKH to modulate sucrose responsiveness of partially fed bees, as they disposed from reserves to be eventually mobilized by AKH treatment. We first studied sucrose responsiveness to pure sucrose solutions. We stimulated the antennae of harnessed bees with six increasing concentrations of pure sucrose solution^48,49^ and recorded PER upon antennal stimulation.

**Figure 5:**
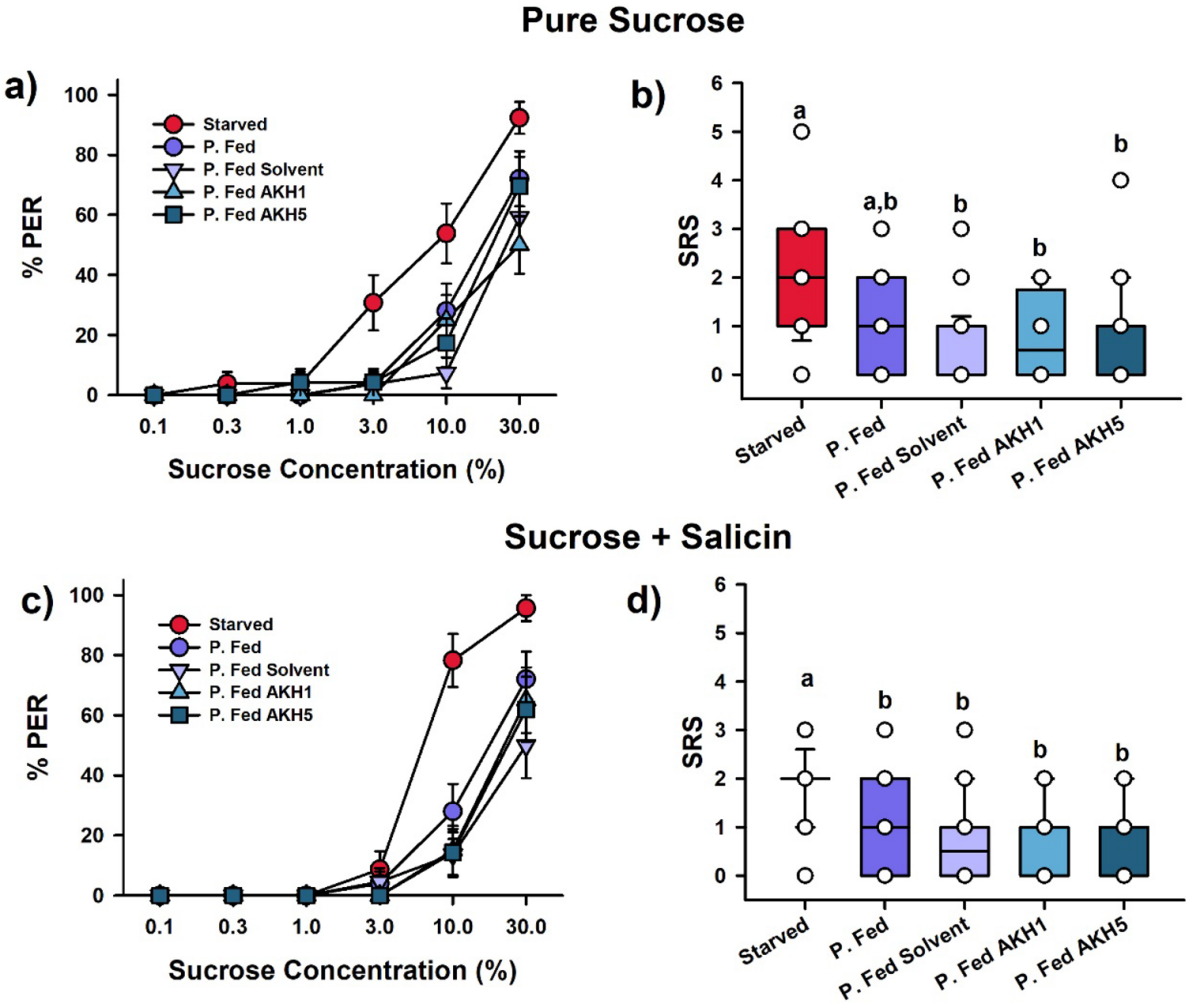
Sucrose responsiveness of partially fed (‘P. Fed’) foraging bees under AKH treatment. Population responses (**a,c**) and individual sucrose responsiveness scores (**b,d**). Bees were either starved (‘Starved’) or partially fed with a honeydew diet. Partially fed bees were left untreated (‘P. Fed’), topically exposed with solvent (‘P. Fed solvent’) or with AKH (either 1 or 5 µg; ‘P. Fed AKH1’ and ‘P. Fed AKH5’, respectively). (**a**) Cumulative proportions of bees showing PER (proboscis extension response) when presented with six pure sucrose solutions of increasing concentration (0.1%, 0.3%, 1%, 3%, 10% and 30% w/w). The percentage of starved bees (n = 26) responding to the three highest sucrose concentrations was higher, yet not significant (P = 0.05), than those of all P. Fed groups (P. Fed, n = 21; P. Fed Solvent, n = 27; P. Fed AKH1, n = 28; P. Fed AKH5, n = 23). (**b**) Individual sucrose response scores (SRS) of bees responding to six pure sucrose solutions of increasing concentration (0.1%, 0.3%, 1%, 3%, 10% and 30% w/w). The figure shows the median (horizontal line) and the 10th, 25th, 75th and 90th percentiles as vertical boxes with error bars and the corresponding overlaid scatter plots. SRS values ranged between six (bees responding to all six concentrations) and 0 (bees not responding to any concentration). Starved bees had significantly higher scores than P. Fed solvent and P. Fed AKH bees. P. Fed bees had intermediate SRS values. Different letters above box plots indicate significant differences (P < 0.05). (**c**) Cumulative proportions of bees showing PER (proboscis extension response) when presented with six solutions of sucrose of increasing concentration (0.1%, 0.3%, 1%, 3%, 10% and 30% w/w) to which 100 mM salicin was added. Starved bees (n = 23) responded more than P. Fed (n = 25) and P. Fed AKH1 bees (n = 20) (P < 0.05 in both cases) for the two highest sucrose concentrations spiked with salicin (10 and 30%). The other comparisons involving starved bees, P. Fed Solvent bees (n = 22) and P. Fed AKH5 bees (n = 21) were not significant. (**d**) Individual sucrose response scores (SRS) of bees responding to six pure sucrose solutions of increasing concentration (0.1%, 0.3%, 1%, 3%, 10% and 30% w/w) spiked with 100 mM salicin. The figure shows the median (horizontal line) and the 10th, 25th, 75th and 90th percentiles as vertical boxes with error bars and the corresponding overlaid scatter plots. Starved bees had significantly higher scores than all fed bees. Different letters above box plots indicate significant differences (P < 0.05). Overall, no effect of AKH on sucrose responsiveness could be detected.

Figure 5a shows the population responses depending on feeding state and topical treatment. The variation observed in the percentage of bees responding to sucrose solutions was marginally non-significant (GLMM, treatment: χ^2^ = 9.41, df: 4, P = 0.052; sucrose concentration: χ^2^ = 9.38, df: 4, P = 0.052). Yet, bees tended to respond more to sucrose solution of higher concentrations, and this increase was more evident in starved bees stimulated with the three highest sucrose concentrations (red circles; 3%, 10% and 30%). Only the comparison between starved bees and partially fed bees treated with the solvent was significant (Dunnett’s post hoc test: Z value = 2.97, P < 0.05).

To analyze responses at an individual level, we quantified for each bee its sucrose responsiveness score (SRS; Fig. 5b), which is defined as the number of sucrose concentrations of pure sucrose solution to which a bee actually responded^53^. Sucrose responsiveness scores varied significantly between groups (Kruskal–Wallis ANOVA by Ranks, df: 4, H = 20.02, P < 0.001) (Fig. 5b). Compared to partially fed bees, starved bees (Fig. 5b; red box) had significantly higher SRS. Yet, the comparison between the SRS of starved and untreated P. Fed bees did not reach significance (U = 195.5, Zadj = 2.55, P = 0.01; alpha level P = 0.005). Starved bees had nevertheless significantly higher SRS than the other groups of treated bees (Starved vs. P. Fed Solvent: U = 145.5, Zadj = 3.87, P = 0.0001; Starved vs. P. Fed 1 µg AKH: U = 168, Zadj = 3.52, P = 0.00044; Starved vs. P. Fed 5 µg AKH: U = 161, Zadj = 2.51, P = 0.004). All comparisons between partially fed groups were non-significant. These results indicate that the topical treatment decreased SRS per se, irrespectively of the substance used and that starved bees tended to have a higher SRS than partially fed bees, as expected. However, no effect of AKH per se on sucrose responsiveness could be detected.

We then tested another group of bees with six concentrations of sucrose solution spiked with salicin, to determine if AKH increased PER towards the unpalatable mixture (Fig. 5c,d). In terms of the population response (percentage of bees responding; Fig. 5c), the pattern of results was similar to that observed with pure sucrose solution (compare with Fig. 5a). Yet, responses now varied significantly according to both sucrose concentration and treatment (Fig. 5c; GLMM, treatment: χ^2^ = 18.19, df: 4, P < 0.01; sucrose concentration: χ^2^ = 14.87, df: 4, P < 0.01). Bees in all groups responded more to the higher concentrations of sucrose solution with salicin. This increase of responses was more evident in starved bees upon stimulation with the two highest sucrose concentrations (red circles; 10% and 30%). The dual comparisons between starved and partially fed bees (Z value = 2.99, P < 0.05) and between starved and partially fed bees treated with 1 µg AKH (Z value = 3.30, P < 0.05) were both significant. The other comparisons involving starved bees were marginally non-significant (vs. fed bees treated with solvent: Z value = 2.66, P = 0.06; vs. fed bees treated with 5 µg AKH: Z value = 2.52, P = 0.08). All other comparisons were non-significant.

Sucrose responsiveness scores varied significantly between groups (Fig. 5d; Kruskal–Wallis ANOVA by Ranks, df: 4, H = 27.74, P < 0.001). Starved bees (red box) had again the highest SRS, which differed significantly from those of all other groups (vs. untreated fed bees: U = 136.5, Zadj = 3.33, P = 0.0009; vs. fed solvent: U = 77.5, Zadj = 4.19, P = 0.00003; vs. fed 1 µg AKH: U = 72.5, Zadj = 4.09, P = 0.00004; vs. fed 5 µg AKH: U = 73, Zadj = 4.21, P = 0.00003). All other comparisons were non-significant, thus showing that treatment with AKH did not affect responsiveness to a sucrose solution spiked with salicin. Starved bees were more prone to accept the mixture between sucrose and salicin, as expected, but AKH did not increase this acceptance.

## Discussion

We analyzed the effect of AKH on both feeding and gustatory processes of starved and partially fed honey bee foragers stimulated or offered with pure sucrose solution or sucrose solution mixed with salicin. The salicin–sucrose solution was used as it has reduced appetitive attraction for honey bees, as confirmed by our results, and thus allowed determining if AKH increases the propensity of bees to consume this unpalatable mixture, as reported for fruit flies^16,17^. Our results show that AKH did affect neither ingestion of sucrose solution (Figs. 3, 4) nor sucrose responsiveness (Fig. 5) and that only the energy budget, which differed between starved and fed bees (Fig. 2), was determinant of higher or lower sucrose responsiveness and ingestion. Starved foragers had higher SRS (Fig. 5) and consumed more sucrose solution than fed foragers (Figs. 3, 4). In addition, no effect of AKH on the proclivity to respond appetitively and ingest an unpalatable mixture of sucrose and salicin was detected (Figs. 3, 4, 5).

### AKH and food ingestion

Our results showed no effect of AKH treatment on ingestion behavior of starved (Fig. 3) and partially fed forager bees (Fig. 4). This absence of effect was predictable in the case of the starved foragers. A decrease in abdominal lipids precedes the onset of foraging^57^ and a significant reduction of the fat body is associated with the foraging state^22^. Foragers thus dispose from less energetic resources to be mobilized from that physiological source^23^. To fuel their foraging flights, active foragers ingest minute quantities of honey stored in the colony^21,58^, which they carry in their crop and consume to reach the exploited food sources^59^. Thus, in starved foragers, captured empty upon arrival at an artificial source and kept for an additional hour without access to food, it could be predicted that AKH would not have a significant effect on food ingestion and sucrose responsiveness as these bees did not have cumulated energetic resources to compensate for starvation. Given their empty crop, low glycogen reserves and reduced fat body^22^, a functional AKH would have no effect on these bees. Food ingestion would be mainly determined by the hunger state of these starved foragers. Note that in the absence of a functional AKH, the same result would be observed, i.e. ingestion behavior would be determined by the hunger state of the bees, even upon an artificial increase of AKH levels. This was precisely the main result obtained in our experiments on starved foragers (Fig. 3). We showed that topical exposure to AKH did not affect the ingestion behavior of these bees, either because it is not functional or because they lacked from enough energetic resources to mobilize from an empty crop and in the absence of a developed fat body. The only factor introducing a difference in ingestion behavior was the feeding state of the forager bees: as expected, partially fed foragers ingested less sucrose solution than starved bees.

Food ingestion experiments with partially fed foraging bees are particularly relevant as these bees disposed from energetic resources in their crop, which could be mobilized if AKH were functional. The results of these experiments (Fig. 4) showed a lack of effect of AKH, irrespective of the dose and time post topical treatment. We found—as expected—that starved foragers consumed more sucrose solution than partially fed foragers (Fig. 4a, groups 30 and 90 min) but no effect of AKH *per se* was detected. This absence of effect is in line with the hypothesis positing that this hormone has lost its effect in foraging bees so that it does not act any longer as the equivalent of glucagon^60^. The same absence of effect was observed for the mixture of sucrose and salicin, which decreased per se the consumption level of both starved and fed bees compared to pure sucrose solution due to the devaluating effect of salicin. Taken together, these results show that AKH does not affect the internal level of energetic resources in foragers so that no changes of ingestive behavior occur upon its manipulation.

### AKH and sucrose responsiveness

Based on the above considerations about the pertinence of using partially fed foragers for these experiments, we determined if AKH modulated the sucrose responsiveness of these bees. We reasoned that if AKH acts as the equivalent of glucagon, it should reduce sucrose responsiveness beyond the level defined by the actual energy budget of the bees. Sucrose responsiveness was evaluated via the proboscis extension response (PER), a reflexive response to the contact of sweet solutions with antennal sucrose receptors on the antennae^37,38^ that reveals the subjective evaluation made by a bee on a reward perceived via this stimulation^61^.

Our results show that AKH had also no effect per se on sucrose responsiveness (Fig. 5). Contrary to biogenic amines such as octopamine and tyramine, which clearly modulate appetitive responsiveness^62,63^, AKH had no effect on sucrose responsiveness scores (SRS). Not surprisingly, PER levels varied between fed and starved bees, being higher in the latter (Fig. 5). Yet, no specific effect of AKH was found besides this difference. Our results are, therefore, consistent with the suggestion that AKH might have lost its original function in honey bee foragers^29^.

A recent study focused on AKH in honey bees and on its possible variation throughout different ages and feeding states^64^. As in the present work, fed and starved bees were subjected to the standard test of sucrose responsiveness^64^ in which PER to increasing sugar concentrations was quantified. As in our experiments, PER levels were higher in starved than in fed bees. They also varied with age, being higher in older foragers compared to nurses^63,65^. Yet, in this work, there was no correlation between increased sucrose responsiveness and AKH levels, which remained constant throughout the ages tested and the feeding regime experienced by the bees^64^. Here we used a different strategy that consisted in manipulating AKH levels and studying the effect of this manipulation on partially fed bees. Overall, the results of these different experiments are coincident: the physiological action of AKH seems to have been lost in foraging bees.

### AKH and the response to an unpalatable mixture of sucrose solution and salicin

In accordance with this conclusion, AKH had no effect per se on both sucrose responsiveness and food ingestion of sucrose solution spiked with salicin. Our ingestion results showed that this mixture was less palatable than pure sucrose solution, as both starved and partially fed bees reduced their consumption when presented with it (see Figs. 3, 4). In fruit flies, bitter solutions are also rejected if the insects are unstarved. Yet, upon starvation, flies accept these substances which they would otherwise reject^16,17^. Neuroegenetic experiments on *Drosophila* flies showed that the mechanism underlying this acceptance involves a molecular cascade in which AKH has a triggering role and which leads to the inhibition of bitter receptors by GABA. As a consequence, flies cannot sense bitter compounds such as salicin and consume them with increasing hunger^16^.

In our experiments, starved bees responded more to sucrose solution spiked with salicin, in particular when the proportion of salicin within the mixture decreased (i.e. for the higher sucrose concentrations; see Fig. 5c,d as an example). Yet, sucrose responsiveness was not affected by AKH and bees did not enhance their responsiveness to sweet-bitter mixtures after topical application of AKH (Fig. 5d). A similar lack of effect was observed in the ingestion of sucrose solution spiked with salicin (Fig. 4b). This shows that AKH does not drive enhanced acceptance of unpalatable substances, either because it does not participate in a cascade modulating peripheric responses to these substances or because the targets of this cascade, i.e. the bitter receptors^16^, are not available in bees. The existence of bitter-receptor homologues of those available in fruit flies has not been demonstrated in bees^32^, and various lines of evidence have questioned the existence of these receptors in these insects^30,32,35,66,67^. The alternative explanation proposed to account for a decrease of responsiveness towards mixtures of sucrose solution and bitter subtances relies on the denaturation of the original taste of the sucrose solution and/or the inhibition of sucrose receptors by these substances^33,68,69^.

Irrespectively of the mechanism by which attraction to the mixture of sucrose and salicin is reduced, AKH does not seem to be involved in this reduction as higher levels of AKH should result in enhanced responsiveness to the sucrose-salicin mixture, which was never observed. Our results thus highlight the diversity of taste and ingestion processes and their underlying molecular cascades in insects. AKH takes part of a cascade leading to modulation of bitter receptor activity and feeding decisions in fruit flies, yet not in honey bees. This conclusion should not be forgotten when enouncing the “model value” of a reduced number of organisms, which may lead to overlooking the ecological and evolutionary diversity of existing biological mechanisms^70^.

### Hormonal regulation of appetitive responses in honey bees

In mammals, insulin and glucagon play an antagonistic role for regulating blood glucose levels^1,60^. In honey bees, the action of insulin has been studied in relation to the regulation of foraging activities^57,71,72^, but less in relation to sucrose responsiveness or food ingestion levels. For instance, a RNAi-based reduction of expression of the *insulin receptor substrate* (*irs*) gene, which encodes a membrane-associated protein in the insulin/insulin-like signalling (IIS) pathway, decreased worker-bee lifespan as a consequence of an earlier onset of foraging behavior, thus showing that the IIS pathway gene influences behavioral maturation and lifespan in honeybees^73^.

The effect of insulin on appetitive responses has been studied in winter and summer worker bees^74^. Insulin injection rendered winter bees more selective in their sucrose responses (i.e. more responsive to higher sucrose concentrations) but had no effect on summer bees, probably because they had already higher levels of insulin. In summer bees of different ages, the effect of insulin was variable: whereas it increased gustatory responsiveness of younger workers, it had the opposite effect on older bees^75^. In a study using a double knockdown of the genes *ultraspiracle* (*usp*) and *vg*, which encode a putative juvenile hormone receptor and vitellogenin, the knockdown induced a decrease of the gene insulin like peptide 1 (*ilp1*), which encodes one of the proposed ligands of the insulin receptors of the honey bee^76^. This treatment was accompanied by an increase of sucrose responsiveness in 7-day-old bees and by an increase of the AKH receptor gene (*Akhr*) in the fat body^76^. Overall, these results show the complexity of the interactions between insulin and sucrose responsiveness, which may change with age, season, caste and further variation in other endocrine actors.

It has been proposed that older bees progress to foraging because lipid stores decrease with age, which correlates with increasing insulin production^57,72^. This suggests that the insulin/AKH system of bees does not play the same role as the insulin/glucagon system of vertebrates. While AKH seems to have lost its original function in honey bee foragers, the capacity of the insulin/AKH system to mobilize nutrient stores depends on the age and caste considered as well as on the season of the year. This system may thus be relevant for the regulation of the division of labor^71^, and may involve additional mechanisms such as octopaminergic signalling^77^. In honey bee nurses, which are the younger bees in the colony, topical application of octopamine leads to a reduction of the hypopharyngeal glands and a concomitant increase in the levels of hemolymph lipids compared with control bees. In contrast, abdominal lipids did not change in response to octopamine treatment^77^. These results indicate that the octopaminergic pathway has the capacity to mobilize nutrients stored in the hypopharyngeal grands, at least in nurse bees, which is considered as a response to stress^77^. Whether the same results are obtained in foragers, for which octopaminergic signalling plays a substantial role in reward representation^78,79^, remains to be determined.

### AKH loss of function and social life

An essential feature of the social life of honey bees is the concentration of nutritional resources (e.g. honey, pollen) within the central place of the hive^20^. This trait is tightly associated with other essential features such as division of labor, overlap of generations and group integration^80^. Honey bee foragers collect nectar (carbohydrates) and pollen (proteins), which they bring to the hive to ensure individual and collective survival. For foraging activities, honey bees consume small quantities of honey available in their colony stocks before departure, which they stock in their crop for energy needs associated with flight^21,25,58^. In this scenario, and with honey stores in the colony exceeding largely individual needs, AKH may have lost its relevance during social-insect evolution. Accordingly, glycogen stores are smaller in the abdomen of active bees (summer bees) although they would be highly needed during the foraging period^23^. Contrary to solitary insects, which depend on fat body energy stores (lipids and glycogen), foraging bees would not rely on such stores because the concentration of resources within the colony is sufficient to satisfy individual needs and because the crop may be the essential source carbohydrates. In energy-demanding situations, if the level of carbohydrates in the hemolymph decreases, sucrose contained in the impermeable crop can pass to the midgut via contractions of the proventriculus^41,81,82^, where it is metabolized into fructose and glucose that circulate in the hemolymph. This process may be more important in foraging bees than the conversion of glycogen from the fat body into trehalose, which can be found in solitary insects. Note, however, that plastic responses to particular life events may modify the physiology of bees so that nutrient mobilization may also be affected by these events. For instance, short-term food deprivation during larval development causes adult honey bees to become more resilient toward starvation. As a consequence of this food deprivation, 7-day old bees have higher glycogen stores and juvenile hormone titers than non-starved bees^83^. Whether in this particular case this glycogen can be converted into trehalose by AKH is unknown.

The loss of functionality of AKH may thus be a consequence of the evolutionary acquisition of a social life^29^. This suggestion leads to further hypotheses, which could be tested using an across-species comparative perspective: (1) other social hymenopterans such as ants and wasps may exhibit a similar loss of function of AKH despite the presence of *Akh* genes in their genomes and (2) solitary hymenopterans, with less concentration of resources within their nests, should have a functional or partially functional AKH. Addressing these hypotheses may lead to a deeper understanding of the physiological consequences of the evolution of sociality in Hymenopterans.
